# Assessing Lumbar Plexus and Sciatic Nerve Damage in Relapsing-Remitting Multiple Sclerosis Using Magnetisation Transfer Ratio

**DOI:** 10.3389/fneur.2021.763143

**Published:** 2021-11-25

**Authors:** Ratthaporn Boonsuth, Rebecca S. Samson, Carmen Tur, Marco Battiston, Francesco Grussu, Torben Schneider, Masami Yoneyama, Ferran Prados, Antrea Ttofalla, Sara Collorone, Rosa Cortese, Olga Ciccarelli, Claudia A. M. Gandini Wheeler-Kingshott, Marios C. Yiannakas

**Affiliations:** ^1^Nuclear Magnetic Resonance Research Unit, Queen Square MS Centre, Department of Neuroinflammation, University College London Queen Square Institute of Neurology, University College London, London, United Kingdom; ^2^Multiple Sclerosis Centre of Catalonia (Cemcat), Vall d'Hebron Institute of Research, Vall d'Hebron Barcelona Hospital Campus, Barcelona, Spain; ^3^Radiomics Group, Vall d'Hebron Institute of Oncology, Vall d'Hebron Barcelona Hospital Campus, Barcelona, Spain; ^4^Centre for Medical Image Computing, Department of Computer Science, University College London, London, United Kingdom; ^5^Philips Healthcare, Guildford, Surrey, United Kingdom; ^6^Philips Japan, Minatoku, Tokyo, Japan; ^7^Department of Medical Physics and Biomedical Engineering, Centre for Medical Image Computing, University College London, London, United Kingdom; ^8^E-Health Center, Universitat Oberta de Catalunya, Barcelona, Spain; ^9^Department of Brain and Behavioural Sciences, University of Pavia, Pavia, Italy; ^10^Brain Connectivity Research Centre, Istituto di Ricovero e Cura a Carattere Scientifico Mondino Foundation, Pavia, Italy

**Keywords:** magnetic resonance neurography (MRN), magnetisation transfer ratio (MTR), multiple sclerosis, relapsing-remitting multiple sclerosis (RRMS), peripheral nervous system (PNS)

## Abstract

**Background:** Multiple sclerosis (MS) has traditionally been regarded as a disease confined to the central nervous system (CNS). However, neuropathological, electrophysiological, and imaging studies have demonstrated that the peripheral nervous system (PNS) is also involved, with demyelination and, to a lesser extent, axonal degeneration representing the main pathophysiological mechanisms.

**Aim:** The purpose of this study was to assess PNS damage at the lumbar plexus and sciatic nerve anatomical locations in people with relapsing-remitting MS (RRMS) and healthy controls (HCs) *in vivo* using magnetisation transfer ratio (MTR), which is a known imaging biomarker sensitive to alterations in myelin content in neural tissue, and not previously explored in the context of PNS damage in MS.

**Method:** Eleven HCs (7 female, mean age 33.6 years, range 24-50) and 15 people with RRMS (12 female, mean age 38.5 years, range 30-56) were recruited for this study and underwent magnetic resonance imaging (MRI) investigations together with clinical assessments using the expanded disability status scale (EDSS). Magnetic resonance neurography (MRN) was first used for visualisation and identification of the lumbar plexus and the sciatic nerve and MTR imaging was subsequently performed using identical scan geometry to MRN, enabling straightforward co-registration of all data to obtain global and regional mean MTR measurements. Linear regression models were used to identify differences in MTR values between HCs and people with RRMS and to identify an association between MTR measures and EDSS.

**Results:** MTR values in the sciatic nerve of people with RRMS were found to be significantly lower compared to HCs, but no significant MTR changes were identified in the lumbar plexus of people with RRMS. The median EDSS in people with RRMS was 2.0 (range, 0-3). No relationship between the MTR measures in the PNS and EDSS were identified at any of the anatomical locations studied in this cohort of people with RRMS.

**Conclusion:** The results from this study demonstrate the presence of PNS damage in people with RRMS and support the notion that these changes, suggestive of demyelination, maybe occurring independently at different anatomical locations within the PNS. Further investigations to confirm these findings and to clarify the pathophysiological basis of these alterations are warranted.

## Introduction

Multiple sclerosis (MS) has traditionally been regarded as a central nervous system (CNS) disorder, with a combination of autoimmune inflammatory effects, demyelination and axonal degeneration. However, neuropathological, electrophysiological, and imaging studies have demonstrated that the peripheral nervous system (PNS) is also implicated in MS, with demyelination and axonal degeneration reported as the main pathophysiological mechanisms (1–3). Understanding the involvement of the PNS in MS could be invaluable for addressing gaps in clarifying mechanisms of pathology affecting people with MS and could, in turn, influence treatment targets.

Magnetic resonance imaging (MRI), specifically magnetic resonance neurography (MRN), has been used widely in recent years to study the PNS, as it enables early detection and precise localisation of neural tissue damage with high sensitivity. Unlike other imaging modalities, MRN has been shown to detect PNS damage in inflammatory, neoplastic, metabolic and traumatic pathologic conditions (4–6). However, even though MRN can provide excellent visualisation of the peripheral nerves, it is *qualitative* in nature and thus may not adequately disentangle the underlying pathophysiological mechanisms involved in each case; nevertheless, its great structural definition allows lesion quantification and volume assessment that in certain situations, e.g., type 1 and 2 diabetes, have provided clinically-relevant quantitative information (7, 8). MRN may also be used successfully to facilitate the acquisition of *quantitative* MRI (qMRI) biomarkers, which provide more specific information regarding the nerve tissue composition, hence contributing towards a better understanding of the underlying pathophysiological mechanisms involved (3, 9).

Magnetisation transfer (MT) imaging has been used to study the interaction between ‘restricted protons' (i.e., protons bound to macromolecules) and “free protons” in the CNS (10–12). The MT effect can be described numerically in clinical settings by the magnetisation transfer ratio (MTR), which represents a normalised difference between MRI signals obtained with and without MT sensitisation. MTR has previously been demonstrated to be a sensitive index of myelin content in white matter in MS patients, with decreasing values in the acute phase of the disease due to demyelination, and increasing values due to recovery in the presence of remyelination (13, 14). Given the neuropathological evidence that demyelination is the main pathophysiological mechanism involved in the PNS in MS (2, 15–17), the use of MTR, which has been shown to be directly influenced by the myelin content in neural tissue, would be appropriate to study PNS damage in MS.

This study, which acknowledges the previous evidence of extensive pathological changes identified in the lower limbs of people with MS (1, 3, 18, 19), aims to investigate the lumbar plexus and sciatic nerve anatomical locations in people with relapsing-remitting MS (RRMS) and healthy controls (HCs) *in vivo* using MTR in order to determine: (a) the presence of PNS damage at the lumbar plexus and sciatic nerve anatomical locations as compared to HCs and (b) the relationship between MTR measures and clinical scores of physical disability.

## Materials and Methods

### Study Participants

HCs and people with RRMS were recruited for this study, all without a history of neuropathy, extremity pain, hypoesthesia or paraesthesia, diabetes mellitus, alcoholism, or other risk factor for polyneuropathy. Some of the patients in this study were recruited in clinic following briefing about the study by a member of the clinical team. All other study participants were recruited from the MS research database (MSRD) at Queen Square Multiple Sclerosis Centre, which contains details of both HCs and people with MS who have previously consented to be contacted about new research studies. Participants had previously joined the MSRD through their involvement in other MS studies, *via* word of mouth, email and posters at University College London and University College London Hospitals NHS Foundation Trust; some of the HCs in the MSRD were members of staff, university students or attending as carers, family, or friends of research participants in MS studies. The study was conducted in line with the International Conference on Harmonisation Good Clinical Practise (ICH GCP) and was approved by the London—Harrow Research Ethics Committee (05/Q0502/101). Informed consent was obtained from all study participants. Demographic information such as age, sex, body weight, height and body mass index (BMI) was recorded for all participants. All people with RRMS were also assessed by a qualified neurologist using the expanded disability status scale (EDSS) (20).

### MRI Acquisition Protocol

MRI imaging was performed at a single imaging centre using a Philips Ingenia CX 3T MRI system (Philips Medical Systems, Best, Netherlands) with the product 28-channel anterior and posterior coils. The possibility of motion during the scans was minimised using appropriate immobilisation technique involving sandbags and Velcro safety straps, while ensuring that the participants were in a comfortable position; the participants were advanced in the scanner “feet-first” and the entire imaging protocol (i.e., lumbar plexus and sciatic nerve) was acquired in one session that lasted <40 min in total.

### Lumbar Plexus Imaging

For visualisation of the lumbar plexus, the 3D “nerve-SHeath signal increased with INKed rest-tissue RARE Imaging” (SHINKEI) sequence was used to acquire images in the coronal plane (21, 22), which were subsequently used for image segmentation. The parameters used with the 3D SHINKEI sequence were as follows: Repetition time (TR) = 2,200 ms, echo time (TE) = 180 ms, field-of-view (FOV) = 280 × 280 mm^2^, voxel size = 1 × 1 × 1 mm^3^, number of excitations (NEX) = 1 and the turbo spin echo (TSE) factor = 56. The duration of the improved motion sensitised driven-equilibrium (iMSDE) was 50 ms, 81 slices were acquired and the scanning time was 8:52 min. For lumbar plexus MTR imaging, a 3D fast field-echo (FFE) dual-echo was used with identical scan geometry to the 3D SHINKEI acquisition to enable easier overlay of the segmented lumbar plexus masks; the parameters used were as follows: TR = 40 ms, TE1 = 2.5 ms, TE2 = 4.5 ms, flip angle α = 10°, in the presence and absence of Sinc-Gaussian shaped MT saturation pulses with nominal α = 360°, 1 kHz frequency and 16 ms duration, number of slices = 81, voxel size = 1 × 1 × 1 mm^3^, FOV = 180 × 180 mm^2^ and scanning time of 5:56 min.

### Sciatic Nerve Imaging

In all participants the right sciatic nerve only was examined. Both sciatic nerves were first located using the 3D SHINKEI sequence acquired with a large FOV in the coronal plane. This image was subsequently used to facilitate prescription of a high-resolution 2D fat-suppressed T2-weighted and MTR acquisitions through the right sciatic nerve (i.e., upper 1/3 of the thigh) in the axial plane, perpendicular to the longitudinal axis of the nerve, in order to enable segmentation of the sciatic nerve and calculation of the MTR values, respectively. The parameters used for the 3D SHINKEI acquisition were as follows: TR = 2,200 ms, TE = 180 ms, FOV = 300 × 420 mm^2^, voxel size = 1.2 × 1.2 × 2 mm^3^, NEX = 1, TSE factor = 56, iMSDE duration = 50 ms, 170 slices, scanning time of 05:43 min.

The parameters used for the 2D fat-suppressed T2-weighted acquisition were as follows: TR = 5,000 ms; TE = 60 ms, FOV = 180 × 180 mm^2^, voxel size = 0.5 × 0.5 × 4 mm^3^, NEX = 1, TSE factor = 11, 30 slices, scanning time of 08:08 min. MTR was acquired with identical scan geometry to the 2D fat-suppressed T2-weighted acquisition using a 3D dual FFE sequence with TR = 40 ms, TE1 = 2.5 ms, TE2 = 4.5 ms, flip angle α = 10°, in the presence and absence of Sinc-Gaussian shaped MT saturation pulses with nominal α = 360°, 1 kHz frequency and 16 ms duration, number of slices = 30, voxel size = 0.5 × 0.5 × 4 mm^3^, FOV = 180 × 180 mm^2^ and scanning time of 5:56 min.

### Image Analysis

**Figure 1:**
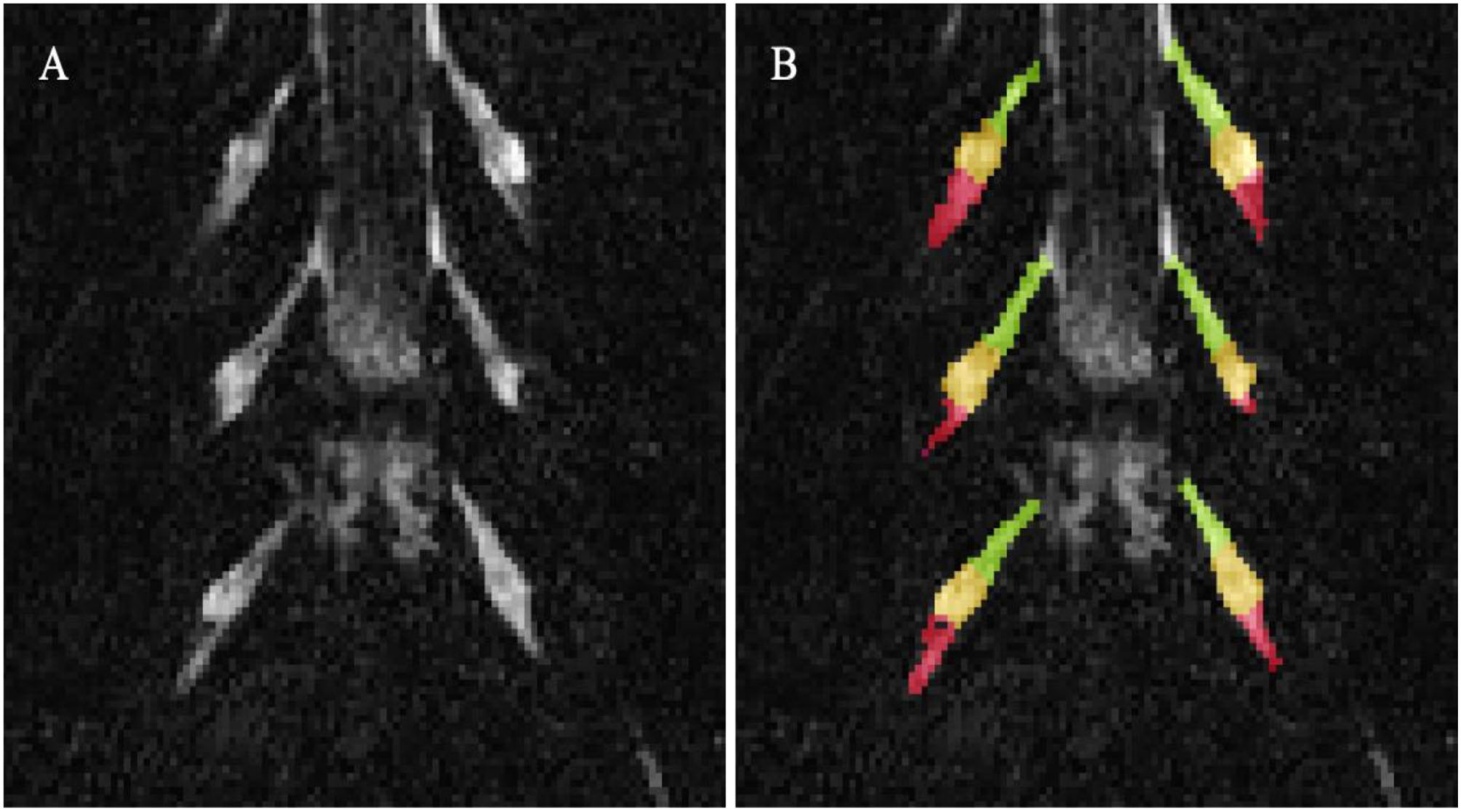
**(A)** Example image obtained using the 3D SHINKEI at the level of the lumbar plexus (L2-L4 segments shown); **(B)** Manual segmentation of the lumbar segments with separate binary masks created for the preganglionic (green), ganglionic (yellow), and postganglionic (red) regions.

**Figure 2:**
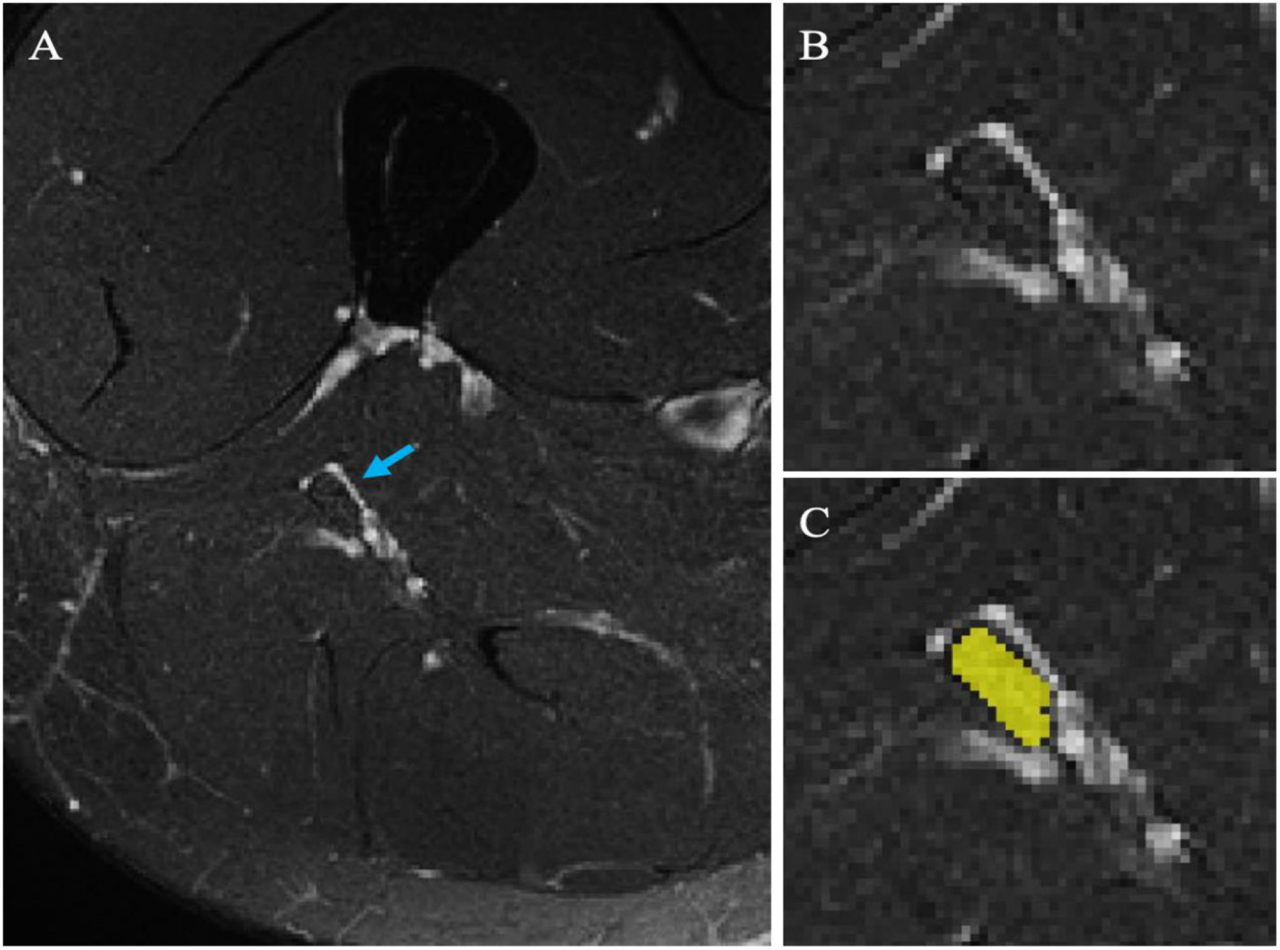
**(A)** Example image of the sciatic nerve (blue arrow) obtained using the high-resolution fat-suppressed T2-weighted sequence; **(B)** Magnified image of the sciatic nerve shown on the left; **(C)** Manual segmentation of the sciatic nerve (binary mask shown in yellow).

Image segmentation was performed manually in FSLview (http://www.fmrib.ox.ac.uk/fsl/). For the lumbar plexus, each lumbar segment (L2-L5) was manually segmented on the 3D SHINKEI images, with separate binary masks created for the preganglionic, ganglionic and post ganglionic regions (Figure 1). The sciatic nerve was segmented manually on fat-suppressed T2-weighted images (Figure 2), with separate binary masks created for each slice. All MTR volumes were co-registered to their respective 3D SHINKEI (plexus) or 2D fat-suppressed T2-weighted volumes (sciatic nerve) using affine registration with NiftyReg (23), to obtain global and region-specific MTR values.

### Statistical Analysis

Statistical analyses were performed using SPSS (IBM, SPSS V27) and STATA/SE 14.2 (StataCorp, College Station, TX). Firstly, differences in demographic factors between HCs and people with RRMS were assessed using the Mann-Whitney U and Chi-square tests as appropriate. Secondly, the Spearman's correlation was used to determine the relationship between MTR measures (at each anatomical location) and demographic factors for each group. Lastly, linear regression models (adjusting for age and sex) were used to: (a) identify differences in MTR values between HCs and people with RRMS at each anatomical location and region, considering the MTR value at each location (one at a time) as the dependent variable and the binary indicator of group (patient vs. HC) as the explanatory variable; (b) identify an association between MTR measures and clinical scores of disability (EDSS), considering the EDSS score as the dependent variable and the MTR value at each location as the explanatory variable.

## Results

### Study Participants

Eleven HCs (7 female, mean age 33.6 years, range 24–50) and 15 people with RRMS without an acute relapse (12 female, mean age 38.5 years, range 30–56) and mean (SD) disease duration of 7.2 (7.5) years were recruited. Table 1 shows the demographic characteristics of all study participants and differences between them, none of which were statistically significant. The median EDSS in people with RRMS was 2.0 (range, 0–3).

**Table 1:** Demographic characteristics of all study participants^*^.

**Parameter**	**Demographic characteristics [mean (±SD)]**		***p***
	**HC (*N* = 11)**	**RRMS (*N* = 15)**	
Age (years)	33.6 (±6.8)	38.5 (±7.9)	0.054
Sex (M/F)	4/7	3/12	0.35
Body weight (Kg)	64.2 (±14.7)	69.9 (±14.9)	0.30
Height (cm)	168.5 (±8.7)	168.6 (±9.3)	0.94
BMI (Kg/m^2^)	22.5 (±3.8)	24.5 (±4.7)	0.22

*HC, healthy controls; RRMS, relapsing-remitting multiple sclerosis; SD, standard deviation; BMI, body mass index*.

* *Differences in sex between groups were assessed using the Chi-square test and differences between groups in all other parameters were assessed using the Mann-Whitney U test*.

### Differences in MTR Values Between HCs and People With RRMS

Mean MTR values were measured for the HC group and people with RRMS at the lumbar plexus and sciatic nerve anatomical locations.

Table 2 shows the results of the Spearman's correlation assessment for each group between MTR measures (at each anatomical location) and demographic factors, none of which were statistically significant. Table 3 shows the summary of all the MTR measurements in HCs and people with RRMS. In the HC group, mean (±SD) MTR in all lumbar segments combined (L2-L5) was 28.0 (±1.7) and in people with RRMS was 28.8 (±1.7). Using a linear regression model, MTR values were not statistically significantly different between the groups when all lumbar segments were combined (*p* = 0.43). In addition, no statistically significant differences in MTR values were identified between the groups in the preganglionic, ganglionic and postganglionic regions, when these were assessed individually.

**Table 2:** Correlations between MTR measures at each anatomical location and demographic characteristics.

**Participant group** **MTR (anatomical region)**	**Spearman's Rho (** ***p*** **)**			
	**Age** **(years)**	**Weight** **(Kg)**	**Height** **(cm)**	**BMI** **(Kg/m^**2**^)**
**HC (*****N*** **= 11)**				
MTR (sciatic nerve)	0.04, *p* = 0.92	−0.31, *p* = 0.36	−0.35, *p* = 0.3	−0.16, *p* = 0.64
MTR (Lumbar plexus)	0.42, *p* = 0.20	−0.09, *p* = 0.79	−0.1, *p* = 0.78	0.06, *p* = 0.87
**RRMS (*****N*** **= 15)**				
MTR (sciatic nerve)	0.21, *p* = 0.46	−0.35, *p* = 0.2	−0.24, *p* = 0.4	−0.38, *p* = 0.16
MTR (Lumbar plexus)	−0.05, *p* = 0.1	−0.01, *p* = 0.1	0.31, *p* = 0.26	−0.23, *p* = 0.42

*MTR, magnetisation transfer ratio; HC, healthy controls; RRMS, relapsing-remitting multiple sclerosis; BMI, body mass index*.

**Table 3:** Summary of all MTR measurements in HCs and people with RRMS at each anatomical location.

**PNS anatomical location**	**MTR Measurement [a.u.] [mean (±SD)]**		***p***
	**HC (*N* = 11)**	**RRMS (*N* = 15)**	
Sciatic nerve	35 (±3.2)	32.4 (±3.6)	0.027^*^
Lumbar plexus	28 (±1.7)	28.8 (±1.7)	0.43
Preganglionic regions	24.7 (±2.8)	26.1 (±3.4)	0.47
Ganglionic regions	28.9 (±2.4)	29.4 (±1.8)	0.39
Postganglionic regions	30.3 (±1.7)	30.8 (±2.5)	0.91

*PNS, peripheral nervous system; MTR, magnetisation transfer ratio; a.u., arbitrary units; SD, standard deviation; HC, healthy controls; RRMS, relapsing-remitting multiple sclerosis*.

* *Indicates statistical significance from linear regression models (adjusting for age and sex) (p < 0.05)*.

**Figure 3:**
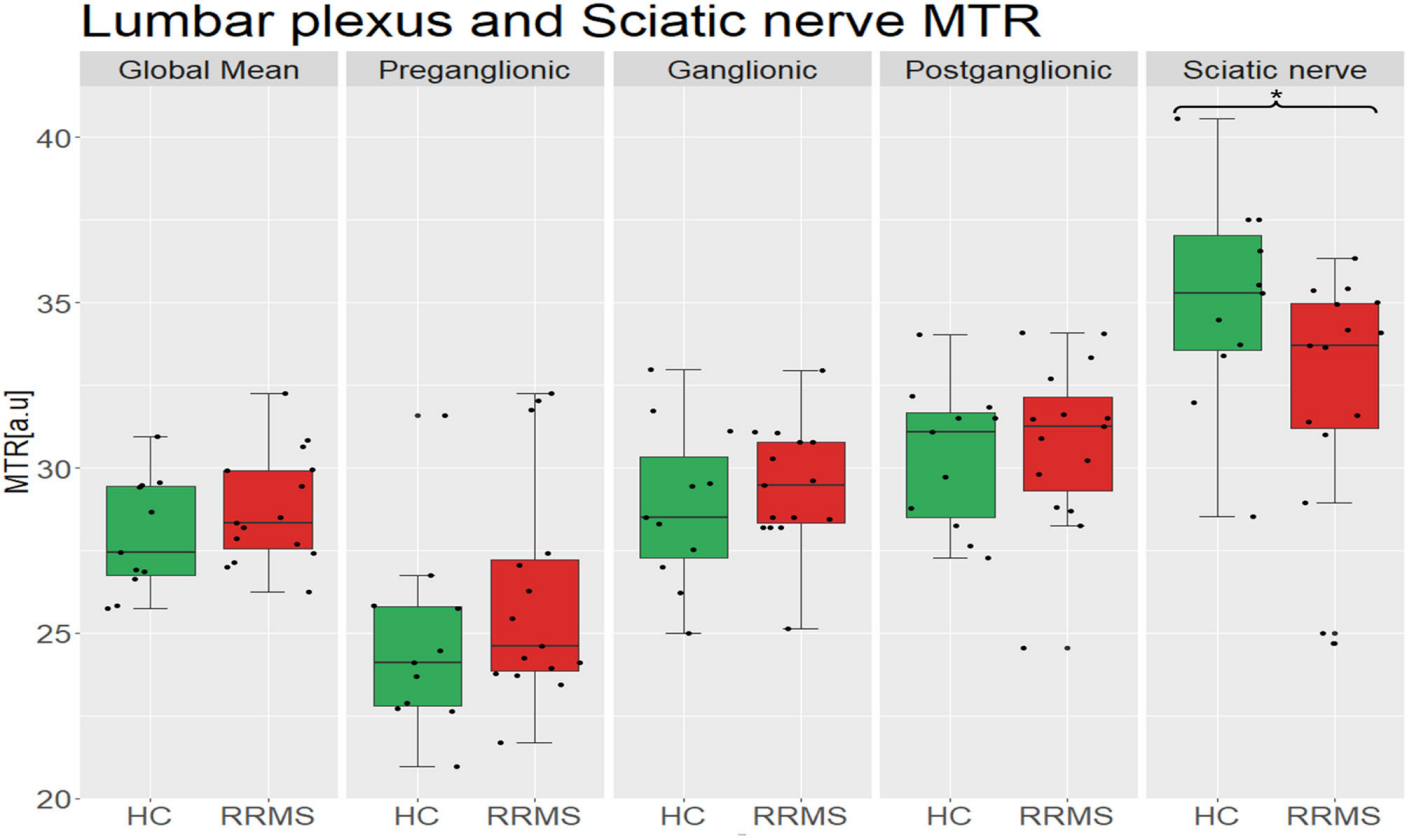
Distribution of MTR values in the lumbar plexus (all lumbar segments combined i.e., global mean) and the sciatic nerve of healthy controls (green) and people with RRMS (red), and also results from separate measurements in the preganglionic, ganglionic and postganglionic regions; no statistically significant differences between the groups were identified in global or regional MTR measurements in the lumbar plexus, but the differences identified in the sciatic nerve were statistically significant (*p* = 0.027). MTR, magnetisation transfer ratio; a.u., arbitrary units; HC, healthy controls; RRMS, relapsing-remitting multiple sclerosis.

In the sciatic nerve, the mean (±SD) MTR value in HCs was 35.0 (±3.2) and in people with RRMS was 32.4 (±3.6); this difference was statistically significant (*p* = 0.027) (Figure 3).

### Correlation Between MTR Measures and Clinical Scores of Disability (EDSS)

Using a linear regression model, no significant associations were identified between the MTR measures and EDSS at any of the anatomical locations investigated.

## Discussion

This study investigated the lumbar plexus and a representative section of the sciatic nerve of people with RRMS and HCs *in vivo* using MTR in order to confirm or rule out the presence of PNS damage at these anatomical locations, and to determine any relationships between MTR measures and clinical scores of disability. The rationale behind this study was the lack of previously reported *in vivo* imaging studies of PNS involvement in MS, coupled with the potential role of MTR in this context, which has previously been demonstrated in the CNS to be directly influenced by the amount of myelin in neural tissue.

MS has traditionally been regarded as a CNS demyelinating disorder, with the majority of previous investigations disregarding the presence, nature and relative contribution of PNS damage to the observed clinical symptoms. However, several studies have reported findings confirming the implication of the PNS in MS (2, 3, 15–17). Specifically, pathological studies have reported demyelinating activity in the peripheral nerves of patients with MS with significant myelin thickness reduction (1). In addition, MRN investigations comparing people with MS and HCs have reported the presence of lesions in the sciatic nerve, tibial, and peroneal nerves, even though the majority of electrophysiological studies were normal (3).

MTR has previously been shown to correlate with myelin content (and, to a lesser extent, axonal damage) in the CNS of people with MS (13). Obtaining MTR measurements in neural tissue, the interaction between “restricted protons” (i.e., protons bound to macromolecules) and “free protons” can be assessed (10, 11). In the context of MTR measurements in PNS, the “restricted protons” maybe demonstrated by the proteins contained in axons and Schwann cells, myelin and collagen tissue (24). MTR could therefore also be advantageous in the investigation of PNS pathology in MS, as it may provide information on nerve damage, collagen integrity and demyelination.

There are only a few studies reporting MTR measurements in the PNS *in vivo*, but neither in people with MS. One study in HCs evaluated the MTR values at different anatomical locations such as the median and foot nerves, demonstrating MTR related differences in the structure and composition of peripheral nerves at different anatomical locations (25). In another study using HCs, the feasibility of obtaining MTR measurements in the lumbar plexus was also demonstrated, opening up the possibilities of studying a wide spectrum of neurological conditions affecting the PNS *in vivo* (9).

In the present study involving people with RRMS, we found no statistically significant differences in MTR values between HCs and people with RRMS when all lumbar segments were combined (L2-L5), nor in the preganglionic, ganglionic and postganglionic regions, when these were assessed individually. One possible explanation may be that this region may not be significantly affected in RRMS but only in more progressive forms of the disease. However, this finding is also likely to be due to the small sample population used in this study or it may be possible that demyelination (or axonal loss) in the lumbar plexus nerves might be subtle and hence not yet detectable through MTR measures.

However, significant differences in MTR values between HCs and people with RRMS were observed more distally at the sciatic nerve of the PNS, which is in agreement with previous neuropathological and imaging reports suggesting the involvement of the PNS in the lower limbs of people with MS (1, 3, 18, 19). MTR reductions in the sciatic nerve in RRMS (but not in the lumbar plexus) identified in this study, represents an interesting finding and supports the notion that pathological changes more distally in the PNS may indeed be independent of CNS changes and even precede more proximal changes, thus providing new insights into mechanisms of disease evolution in MS. Such changes are likely to be due to demyelination, but could also represent axonal loss and inflammation. Previous electrophysiological and imaging studies in MS have suggested that PNS changes may be attributed to Wallerian degeneration caused by spinal cord lesions (26), peripheral codemyelination of the PNS in MS, which is likely to be caused by immunologic reactions and destruction of molecules such as connexin 32 or myelin-associated glycoproteins (3, 19, 27, 28), or epitope spreading, likely to be seen in a special subgroup of people with MS who go on to develop peripheral demyelinating neuropathy during the long course of MS (2). In the present study, electrophysiological assessments and imaging of the relevant spinal cord segments were not included, and as such the interpretation of the findings can only be based on the available data. Given that MTR imaging is a more sensitive index to myelin content than axonal degeneration (13), the possibility of Wallerian degeneration cannot be fully elucidated, although it was previously demonstrated that accounting for spinal cord lesions did not help explain the observed PNS changes in the lower limbs (3). It is therefore conceivable that the significantly lower MTR values identified in people with RRMS in the sciatic nerve (but not the lumbar plexus) in this study could be indicative of peripheral codemyelination, which is also in line with previous findings (3). Further multi-modal investigations may be needed to confirm the pathophysiological basis of the alterations in MTR identified in this study, for example with the use of diffusion-weighted imaging, as demonstrated in previous studies of peripheral neuropathy (29–31).

In the second part of this study, we evaluated the relationship between MTR measures and clinical scores of physical disability. The results showed no relationship between the MTR measures in the PNS and clinical scores of disability (EDSS) for either of the anatomical locations studied in the selected cohort of people with RRMS. There are a number of possible explanations for this observation, including the small sample size, measurement variability or the very low EDSS score of this group of people with RRMS (median EDSS = 2.0) that makes it not ideal to detect associations between MTR and clinical disability. Furthermore, the sensitivity of EDSS in terms of assessing PNS damage is not well-documented and more specialised clinical tools in future investigations are warranted.

### Limitations

This study has a number of limitations, which should be considered when interpreting the results. To begin, the limited sample size may have influenced statistical analyses. However, in a previous study in 10 healthy controls of similar age using the same MTR acquisition, sample-size calculations showed that to detect a 10% change with 80% power at 5% significance in the lumbar plexus, this would require a minimum of 20 subjects (10 patients and 10 controls) (9). Nevertheless, a larger sample size as well as other factors, such as the age range, disease duration and disability range, would have enabled a more complete assessment of pathological changes of the PNS in people with RRMS. Furthermore, the study relied upon accurate tissue segmentation and the effect of partial volume voxels may have affected the interpretation of results. To minimise partial volume effects, we made sure to perform the segmentations in a conservative fashion and segmentations were also independently performed by an additional experienced rater who was blinded to the results of the first rater and the clinical details of the study participants; all the segmentations from the two raters were tested and found to be in good agreement (see Supplementary Material).

Another limitation is related to the long acquisition time, which was the main reason for examining the right sciatic nerve only in this pilot study; a common approach in electrophysiological studies in MS is to examine both sides as a reflection of the global affection of the motor pathways (32–34). However, multi-modal investigations to extract biophysically meaningful imaging biomarkers related to PNS damage are likely to involve even longer acquisitions and this will need to be accounted for in future study designs. Finally, electrophysiological examinations and clinical assessments (in addition to EDSS), such as assessments of grip strength, vibration perception thresholds, Nine Hole Peg Test, Timed 25-Foot Walk Test, Multiple Sclerosis Walking Scale-12 and Modified Ashworth Scale, were not included in this study, and it is conceivable that these may have added value to this investigation.

Future investigations will aim to include additional MS clinical phenotypes in a longitudinal study design involving thorough clinical assessments and electrophysiology studies alongside qMRI, aiming to understand mechanisms of PNS damage in MS and the interdependencies with CNS pathology. Understanding mechanisms of PNS damage in MS and clarifying the relative contribution of this damage to the observed clinical symptoms offers a novel clinical perspective of the MS pathology; the use of quantitative imaging biomarkers sensitive to the underlying pathophysiological mechanisms of such alterations, including demyelination, axonal loss and inflammation provides real potential for tailored management of symptoms, treatment options and prognosis of progression, with possible improved clinical outcomes.

## Conclusions

This study has demonstrated significant reductions in MTR values in the sciatic nerve of people with RRMS as compared to HCs, but no similar changes were identified in the lumbar plexus of the same study participants. This finding demonstrates that pathological changes in myelin content can be present at more distal anatomical locations in the PNS of people with RRMS, but maybe independent of changes at more proximal locations in the PNS, providing further insight into the mechanisms of the pathological changes involved in MS. Furthermore, the study has demonstrated no relationships between MTR measures and clinical scores of disability (EDSS) at any of the anatomical locations in the PNS studied in this cohort of people with RRMS, suggesting that future investigations involving additional MS clinical phenotypes are warranted.

## Data Availability Statement

The raw data supporting the conclusions of this article will be made available by the authors, without undue reservation.

## Ethics Statement

The studies involving human participants were reviewed and approved by London-Harrow research Ethics Committee. The patients/participants provided their written informed consent to participate in this study.

## Author Contributions

MCY and CGW-K contributed to the conception and design of the study. SC organized the database. CT performed the statistical analysis. RB wrote the first draft of the manuscript. RB, MCY, and RSS wrote sections of the manuscript. All authors contributed to manuscript revision, read, and approved the submitted version.

## Funding

The UK MS Society and the UCL-UCLH Biomedical Research Centre for ongoing support. CGW-K receives funding from the MS Society (#77), Wings for Life (#169111), BRC (#BRC704/CAP/CGW), UCL Global Challenges Research Fund (GCRF), MRC (#MR/S026088/1), Ataxia UK. FP had a non-clinical Postdoctoral Guarantors of Brain fellowship (2017-2020). FP was supported by the National Institute for Health Research, UCL Hospitals Biomedical Research Centre. CT is being funded by a Junior Leader La Caixa Fellowship (fellowship code is LCF/BQ/PI20/11760008), awarded by la Caixa Foundation (ID 100010434). She has also received the 2021 Merck's Award for the Investigation in MS, awarded by Fundación Merck Salud (Spain). In 2015, she received an ECTRIMS Post-doctoral Research Fellowship and has received funding from the UK MS Society. She has also received honoraria from Roche and Novartis, and is a steering committee member of the O'HAND trial and of the Consensus group on Follow-on DMTs. This project has received funding under the European Union's Horizon 2020 research and innovation programme under grant agreement No. 634541 and from the Engineering and Physical Sciences Research Council (EPSRC EP/R006032/1), funding FG. FG was currently supported by PREdICT, a study at the Vall d'Hebron Institute of Oncology in Barcelona funded by AstraZeneca.

## Conflict of Interest

FG is supported by PREdICT, a study co-funded by AstraZeneca (Spain). TS is an employee of DeepSpin (Germany) and previously worked for Philips (United Kingdom). MB is an employee of ASG Superconductors. MY is employed by Philips (Japan). AstraZeneca, Philips, DeepSpin and Superconductors were not involved in the study design; collection, analysis, interpretation of data; manuscript writing and decision to submit the manuscript for publication; or any other aspect concerning this work. The remaining authors declare that the research was conducted in the absence of any commercial or financial relationships that could be construed as a potential conflict of interest.

## Publisher's Note

All claims expressed in this article are solely those of the authors and do not necessarily represent those of their affiliated organizations, or those of the publisher, the editors and the reviewers. Any product that may be evaluated in this article, or claim that may be made by its manufacturer, is not guaranteed or endorsed by the publisher.
