# Supplementary material for: Assessing Lumbar Plexus and Sciatic Nerve Damage in Relapsing-Remitting Multiple Sclerosis Using Magnetisation Transfer Ratio
Source: Front Neurol. 2021 Nov 25;12:763143. doi: 10.3389/fneur.2021.763143 (PMC8654928; doi:10.3389/fneur.2021.763143)
Supplement: Supplementary file 1 [file Data_Sheet_1.PDF]

Supplementary Material

Supplementary Data

To assess the reproducibility of manual image segmentation, images from 5 out of 11 HCs were segmented twice by the same rater (‘intra-rater’), with binary masks created on different occasions (at least two weeks apart) for both the lumbar plexus and the sciatic nerve. In order to assess ‘inter-rater’ reproducibility, segmentations were also independently performed by an experienced rater who was blinded to the results of the first rater and the clinical details of the study participants. To ensure that partial volume effects were minimised, the two raters were instructed to perform the segmentations conservatively by avoiding the boundary of the nerve (Figure S1).

All binary segmentation masks from both raters were then used to calculate the dice similarity coefficient (DSC) (37), which is a commonly used spatial overlap index and a reproducibility validation metric. The DSC is a measurement between two datasets and represents the size of the union of two sets divided by the average size of the two sets. The value of DSC ranges from 0, indicating no spatial overlap between two sets of binary masks, to 1, indicating complete overlap. Table S1 shows the DSC results of the ‘intra-rater’ assessment and Table S2 the results of the ‘inter-rater’ assessment.

Supplementary Figures and Tables

Supplementary Table 1. Intra-rater reproducibility

**Intra-rater reproducibility**		
	Dice similarity coefficient (DSC)	
**Subject**	Lumbar plexus	Sciatic nerve
**1**	0.77	0.76
**2**	0.84	0.80
**3**	0.77	0.79
**4**	0.89	0.87
**5**	0.86	0.73
**Mean**	0.83	0.79
**S.D.**	0.05	0.05

Supplementary Table 2. Inter-rater reproducibility

**Inter-rater reproducibility**		
	Dice similarity coefficient (DSC)	
**Subject**	Lumbar plexus	Sciatic nerve
**1**	0.69	0.81
**2**	1.00	0.86
**3**	0.74	0.82
**4**	0.73	0.83
**5**	0.69	0.81
**Mean**	0.77	0.83
**S.D.**	0.13	0.02

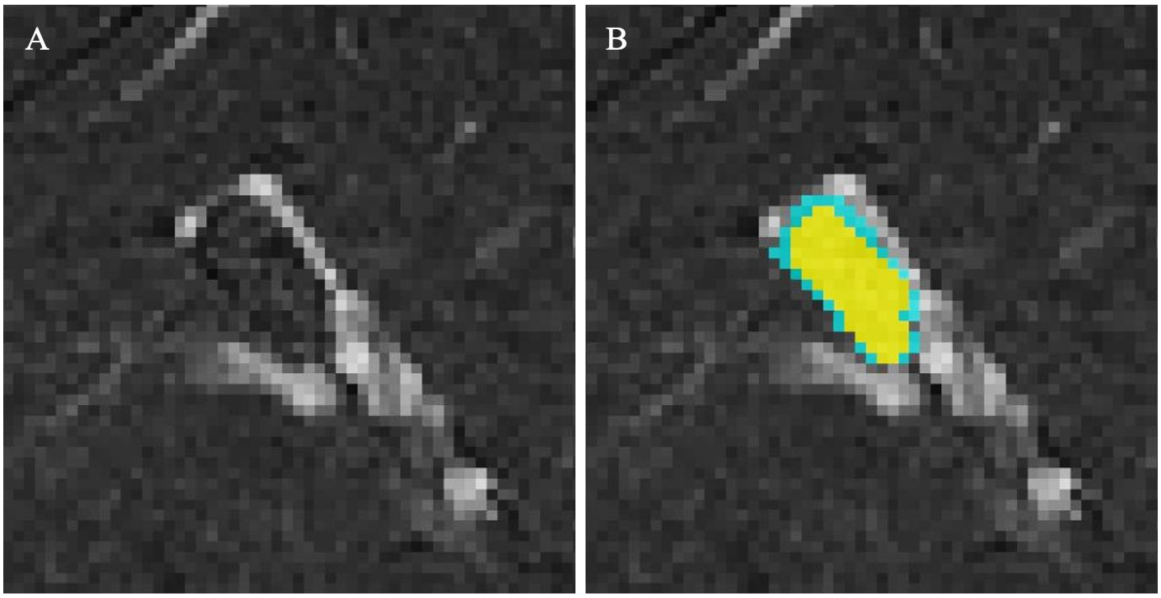

Supplementary Figure 1. A) Cross-section of the sciatic nerve shown on a fat-suppressed T2w image; B) example of conservative image segmentation of the sciatic nerve (yellow colour) avoiding inclusion of the nerve boundary (blue colour).
